# Equilibrist: A Browser-Based Platform for Fitting Equilibrium and Rate Constants from Optical and NMR Data

**DOI:** 10.1021/acs.jcim.6c01004

**Published:** 2026-07-23

**Authors:** Eric Masson

**Affiliations:** Department of Chemistry and Biochemistry, 1354 Ohio University, Athens, Ohio 45701, United States

## Abstract

Equilibrist is a free, open-source platform for the quantitative analysis of chemical equilibria and kinetics. It runs in a local web browser, requires no installation, and uses a plain-text scripting language to specify reversible and irreversible reactions, polyprotic acid–base networks, and thermodynamic-cycle constraints. Five fitting modes cover species concentration, NMR shifts (fast exchange), NMR integrations (slow exchange), a mixed mode, and UV–vis/emission spectrophotometry, optimized by the L-BFGS-B and Nelder–Mead methods. A comprehensive statistical layer adds information criteria (AIC, AICc, BIC) with Akaike weights and a Fisher *F*-test for model selection, residual diagnostics (Durbin–Watson and Shapiro–Wilk statistics, Q-Q plot), and three uncertainty quantification families (bootstrap, jackknife, and Monte Carlo input propagation). Parameter identifiability is mapped by 1D and 2D RMSE profiles, sloppy-spectrum eigen decomposition, correlation heatmaps, *t*-tests, and a novel local sensitivity coefficient ξ. The combined diagnostics let the user move past *R* ^2^ coefficients and Hessian standard errors to assess parameter estimates and the chemical model itself.

## Graphical abstract

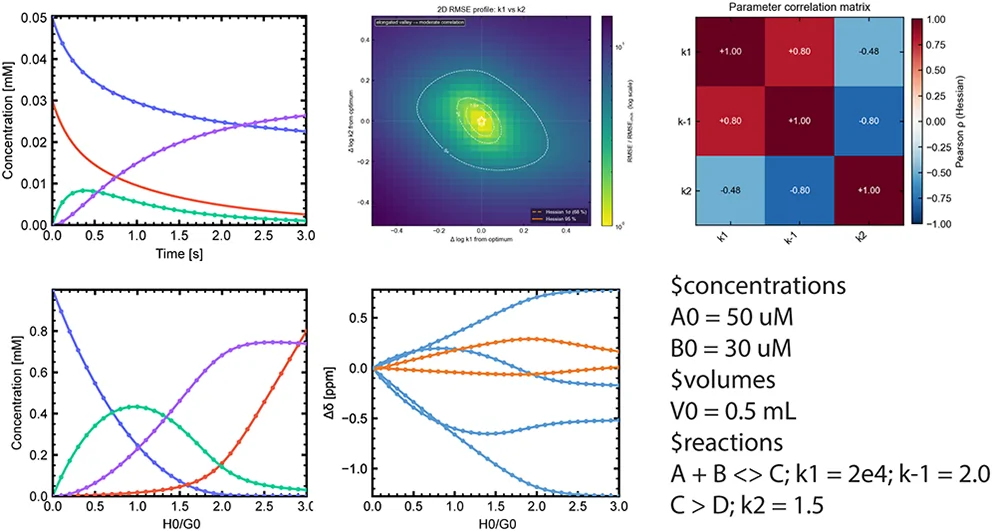

## Introduction

The determination of thermodynamic and kinetic parameters, such as binding constants and rate constants from spectroscopic titration data, is central to modern physical organic chemistry, analytical chemistry, biochemistry, and drug discovery. Despite this fundamental importance, the software landscape remains fragmented: many tools are proprietary, platform-specific, limited to a single data type, or require expert configuration before a measurement can be processed.

Existing solutions address portions of this problem but each with significant restrictions. DynaFit (developed by Kuzmič),^1,2^ the most widely cited program for biochemical kinetics and equilibria, is freely available to academic users but is restricted to Microsoft Windows. The HYPERQUAD suite^3^ (HypSpec, HypNMR, etc.) provides robust algebraic equilibrium solvers for potentiometric, UV–vis spectrophotometric, NMR, and calorimetric titration data; the programs refine thermodynamic equilibrium constants but do not model reaction kinetics, and they are distributed under restricted licenses rather than as freely available software. Thordarson and co-workers established the theoretical and practical framework for NMR and UV–vis titration analysis in supramolecular chemistry^4–6^ and implemented it in Bindfit,^7^ a free browser-based tool hosted at supramolecular.org; Bindfit covers a curated set of stoichiometric models (1:1, 1:2, 2:1), but does not support user-defined reaction networks or kinetics. ReactLab^8^ (developed by Mader) and SupraFit^9^ (developed by Hübler) provide flexible fitting environments for spectroscopic titration and kinetic data. ReactLab is a commercial desktop package capable of modeling arbitrary reaction mechanisms in spectrophotometric data sets, while SupraFit is an open-source Qt-based application supporting supramolecular titration analysis and selected kinetic models. However, neither combines browser-based accessibility with open-source code and multimodal spectroscopic fitting in a single platform. For kinetic simulation in systems biology, COPASI^10^ (developed by Mendes and co-workers) is a widely used open-source tool but provides no spectroscopic fitting. Musketeer, developed by Soloviev and Hunter,^11^ is a freely available, open-source, cross-platform graphical program for fitting titration data from UV–vis, fluorescence, and NMR experiments. It supports user-defined binding models with arbitrary stoichiometries and complex equilibria through a graphical interface, enabling analysis of multicomponent and competitive binding systems. However, Musketeer does not model time-domain kinetics, and it requires a local installation. SIVVU,^12^ developed by Vander Griend and co-workers, is a web-based platform dedicated to the global analysis of UV–vis spectrophotometric titrations; however, it does not currently support NMR data, multimodal global fitting across spectroscopies, or chemical kinetics fitting.

Equilibrist was designed to address these shortcomings by providing a single, unified platform that (1) runs in a standard web browser without installation of specialty software beyond a Python environment, (2) accepts any combination of preequilibria, slow equilibria, and irreversible reactions expressed in an intuitive scripting language, (3) allows the user to manipulate conditions and reactions in the browser window and to view impacts on theoretical concentrations, (4) supports concentration, NMR (fast, slow, and hybrid regimes), and UV–vis or fluorescence spectroscopy input data, (5) enforces physical constraints through a flexible penalty-based system, (6) produces output suitable for direct inclusion in presentations and manuscripts, and (7) returns robust statistical analysis. The browser-based architecture, enabled by the Streamlit framework,^13^ provides an interactive graphical interface that updates in real time as the user modifies parameters, and all computations run locally.

In the following sections, we describe the theoretical basis of the thermodynamic and kinetic solvers, the parameter estimation framework, the constraint formalism, and the user interfaces. We then illustrate the capabilities of the software with a set of representative case studies.

## Theoretical Background

### Thermodynamic Solver

For a system containing *N* species, the equilibrium constant *K* for reaction *j* is defined by eq 1.

1 $$K_{j}=\prod_{i}{\left[X_{i}\right]}^{n_{\mathrm{ij}}}$$

where *n*_*ij*_ is the stoichiometric coefficient of species *i* in reaction *j* (positive for products, negative for reactants). The concentrations at equilibrium are found by solving a system of nonlinear equations comprising the mass action expressions and the mass balance constraints. Equilibrist employs an iterative Newton–Raphson method in log-concentration space, which enforces positivity of all concentrations automatically. The solver operates on the set of free species concentrations and recovers all dependent species from the equilibrium expressions analytically at each iteration.

### Kinetic Solver

When any kinetic step is declared, Equilibrist uses a time-domain ordinary differential equation (ODE) solver. The concentration vector **c**(*t*) is obtained from eq 2.

$$\frac{dc}{\mathrm{dt}}=S\cdot r\left(c,k\right)$$ 2

where **c**(*t*) is the vector of species concentrations, **S** is the stoichiometric matrix, **r**(**c**, **k**) is the vector of elementary rate expressions, and **k** is the vector of rate constants. Integration is performed with the Radau stiff-solver,^14^ which is appropriate for the stiff systems that arise when fast equilibria coexist with slow kinetic steps. Preequilibria are implemented by assigning large reversible rate constants.

### Parameter Estimation

All fitting modes minimize an objective function *F*(**θ**) comprising the sum of squared residuals (SSR) between predicted and observed quantities *y*_*i*_^o^ and *y*_*i*_, plus a penalty term encoding constraints *P*(**θ**, C) (see eqs 3–5)­

3 θ=log⁡K

4 $$F\left(\theta \right)=\sum_{i}{\left(y_{i}^{o}-y_{i}\right)}^{2}+P\left(\theta ,C\right)$$

5 $$P\left(\theta ,C\right)=\sum_{c}\omega_{c}f_{c}{\left(\theta \right)}^{2}$$

where the sum runs over all declared constraints *c*, ω_*c*_ is the penalty weight for constraint *c*, *f*_*c*_(**θ**) is the signed deviation from the target value of constraint *c*, and *f*_*c*_(**θ**) is replaced by max­(0, *f*_*c*_(**θ**)) for inequality constraints so that the penalty vanishes within the allowed region.

Equilibrist employs a sequential two-method strategy. The L-BFGS-B method^15^ is applied first and uses gradient information for rapid convergence in well-conditioned problems. If L-BFGS-B does not improve the objective relative to the starting point, which can occur when equilibrium constants are poorly determined by the data or when constraints create sharp boundaries, the Nelder–Mead method^16^ is applied subsequently as a derivative-free alternative. Both methods can be individually enabled or disabled by the user.

Parameter uncertainties are estimated from the Hessian of the objective function evaluated at the best-fit parameter values, using only the data residuals and excluding constraint penalties. This ensures that the reported errors reflect the information content of the experimental data, and are not artificially reduced by the penalty weights ω_*c*_. The covariance matrix **∑** is approximated using eqs 6 and 7.

6 $$\Sigma =\sigma^{2}{\left(J^{T}J\right)}^{-1}={2\sigma }^{2}{\left(H^{\mathrm{data}}\right)}^{-1}$$

7 $$\sigma^{2}=\frac{\sum_{i}{\left(y_{i}^{o}-y_{i}\right)}^{2}}{n-p}$$

where **J** is the Jacobian matrix of residuals, σ^2^ is the residual variance, *n* is the number of data points, *p* is the number of fitted parameters, and **H** ^data^ is the Hessian matrix of the second derivatives of the SSR objective function with respect to the fitted parameters, evaluated numerically at the best-fit solution. The square roots of the diagonal elements of **∑** give the standard error on each parameter, reported alongside the fitted value.

### Fitting Options

Equilibrist supports three fitting options; they are available for the fitting of both equilibrium and rate constants, and each can be used with or without active constraints:

(1) Concentration mode. Theoretical species concentrations computed by the thermodynamic or kinetic solvers are compared directly to the measured concentration data.
(2) NMR mode. (a) For fast-exchange systems, the observed chemical shift δ_obs_ is a population-weighted average (see eq 8), where *x_i_* are the mole fractions of each species and δ*_i_* are the chemical shifts of the pure species:
8 $$\delta_{\mathrm{obs}}=\sum_{i}x_{i}\delta_{i}$$
  ­(b) For slow-exchange systems, absolute concentrations are obtained from signal integrations. (c) In hybrid regimes (with concomitant fast and slow exchanges), integration and chemical shift residuals are combined into a single weighted objective function, allowing simultaneous analysis of both exchange regimes.
(3) UV–vis/fluorescence spectral mode. At each trial value of the parameter vector **θ** proposed by the optimizer (see eqs 3–5), the thermodynamic or kinetic solver fully determines the species concentration matrix **C** (number of spectra *n*_*S*_ × number of absorbers *n*_*A*_), and the measured absorbance matrix **A** = **C**·**ε** is then linear with the molar absorptivity matrix **ε** (with the path length embedded in the matrix). Equilibrist exploits this by solving for **ε** analytically at each step as the non-negative least-squares solution to **A** = **C**·**ε**, so that the nonlinear optimization is performed over **θ** alone. This variable projection approach^17^ reduces the dimensionality of the nonlinear search from *n*_θ_ + *n*_*A*_ *n*_λ_ to *n*_θ_, where *n*_θ_ and *n*_λ_ are the total number of fitted parameters and wavelengths, respectively. This substantially improves convergence speed. When the concentration profiles of two or more absorbing species are nearly proportional across the titration series, their individual contributions to the absorbance cannot be separated from the spectral data alone; only the product of the equilibrium constants can be determined. Equilibrist detects this condition by computing the condition number κ­(**C**) of the concentration matrix **C** (see eq 9):
9 $$\kappa \left(C\right)=\frac{\sigma_{\max}}{\sigma_{\min}}$$
  where σ_max_ and σ_min_ are the largest and smallest singular values of **C**, respectively; when two or more concentration profiles are nearly proportional, the columns of **C** become nearly linearly dependent, σ_min_ collapses toward zero, and κ­(**C**) diverges. If κ­(**C**) > 10^4^, Equilibrist displays a warning identifying the best-determined parameter combination, often the cumulative binding product ∏_*i*_ *K*_*i*_, and alerts the user that the individual constants cannot be reliably extracted from the data as collected.

### Statistical Validation

The Hessian-based standard errors of eq 7 assume that the residuals are independent and normally distributed with constant variance across the experiment, that the best-fit parameters lie within their imposed bounds rather than against them, and that the sum-of-squared-residuals surface is well approximated by a parabolic well near the optimum. When any of these conditions fail (for example, when consecutive residuals are autocorrelated or when the residual distribution is skewed or heavy-tailed), the Hessian errors will systematically under- or overestimate the true uncertainty. Equilibrist therefore complements the Hessian standard errors with a series of additional statistical diagnostics in a combination not currently offered by any single existing package.

Equilibrist proposes a set of information criteria for model selection. For models fitted by least squares under a Gaussian-noise assumption, the maximized log-likelihood $l_{\max}$ is obtained from eqs 10 and 11, where *n* is the number of data points entering the fit, *r*_*i*_ is the *i*-th residual, and *S* is the sum of squared residuals at the optimum.

10 $$S=\sum_{i}r_{i}^{2}=\sum_{i}{\left(y_{i}^{o}-y_{i}\right)}^{2}$$

11 $$l_{\max}=-\frac{n}{2}\ln\frac{S}{n}$$

Akaike’s information criterion (AIC; see eq 12),^18^ its bias-corrected variant AIC_c_,^19^ as well as the Bayesian information criterion of Schwarz (BIC)^20^ are obtained from eqs 12–14, where *p* is the number of fitted parameters.

12 $$\mathrm{AIC}=n\ln\frac{S}{n}+2p$$

13 $${\mathrm{AIC}}_{c}=\mathrm{AIC}+\frac{2p\left(p+1\right)}{\left(n-p-1\right)}$$

14 $$\mathrm{BIC}=n\ln\frac{S}{n}+p\ln n$$

When two or more candidate reaction networks are fitted to the same data set, Equilibrist returns Akaike weights ω_*i*_ for each scenario *i* from eq 15, i.e., the relative likelihood that candidate *i* is the best approximation to the process.^21^

15 $$\omega_{i}=\frac{e^{-\Delta {\mathrm{AIC}}_{i}/2}}{\sum_{j}e^{-\Delta {\mathrm{AIC}}_{j}/2}}$$

Equilibrist then computes the Durbin–Watson statistic *d* (see eq 16),^22^ which tests for neighbor-to-neighbor autocorrelation in the ordered residual sequence (i.e., the tendency for adjacent residuals *ri* and *r*_*i*–1_, in consecutive aliquot addition, time step, or wavelength, to share the same sign). Values near 2 are consistent with the independent residual assumption that underlies the Hessian standard errors of eq 7, whereas *d* significantly below 2 indicates a smooth trend left in the residuals, often indicative of an under-parametrized model, a baseline drift, or a missing equilibrium.

16 $$d=\frac{\sum_{i=2}^{n}{\left(r_{i}-r_{i-1}\right)}^{2}}{S}$$

Equilibrist also computes the Shapiro–Wilk *W* statistic with its associated *p*-value^23^ as a test of residual normality. *W* lies between 0 and 1; values close to 1 are consistent with normally distributed residuals, while a small *p*-value (typically below 0.05) signals a departure from normality and warns that the Hessian standard errors of eq 7, which presuppose Gaussian residuals, may under- or overestimate the true parameter uncertainty.

Equilibrist presents a figure with residuals versus the experimental predictor, a residual histogram, a normal Q–Q plot, and a metrics table with the parameters described above. The figure can be supplemented with a parameter profile panel that implements the one-dimensional RMSE profile diagnostic of Musketeer^11^ and is a direct test of parameter identifiability.^24,25^ Each fitted parameter θ_*k*_ is pinned in turn within a range of values spanning around its fitted optimum; every other free parameter is refit at each point within the range, and the normalized root-mean-square error (RMSE/RMSE_opt_) is plotted as a function of Δlog θ_*k*_. The shape of the resulting curve is diagnostic: a sharp V-shaped valley confirms that θ_*k*_ is well-determined by the data, while a flat profile signals that θ_*k*_ is unidentifiable given the other free parameters. An example illustrating these features is proposed below (see Figures 1 and 2 and associated narrative).

**Figure 1:**
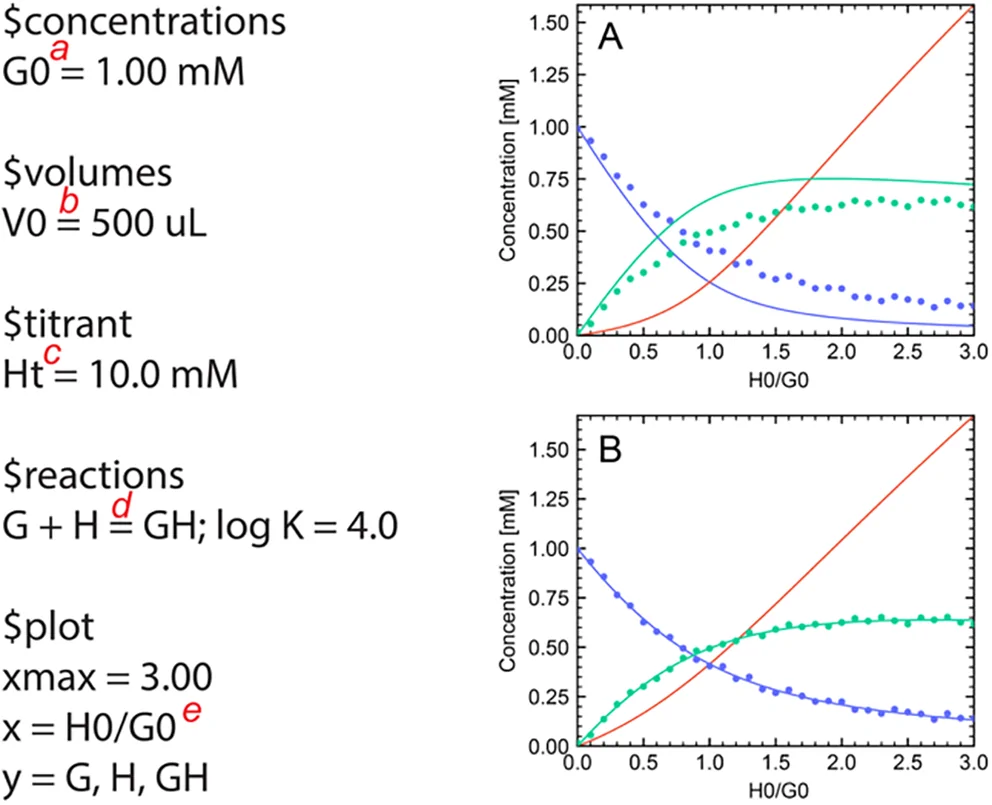
Equilibrist script for a 1:1 host–guest binding process with a binding constant *K*. Plots generated by Equilibrist: concentration of free guest **G** (blue trace), free host **H** (red trace), and host–guest complex **GH** (green trace) as a function of *H*_0_/*G*_0_, with (A) log *K* = 4.0; experimental data points before fitting, and (B) log *K* = 3.46 (±0.01); traces and data points after fitting.

**Figure 2:**
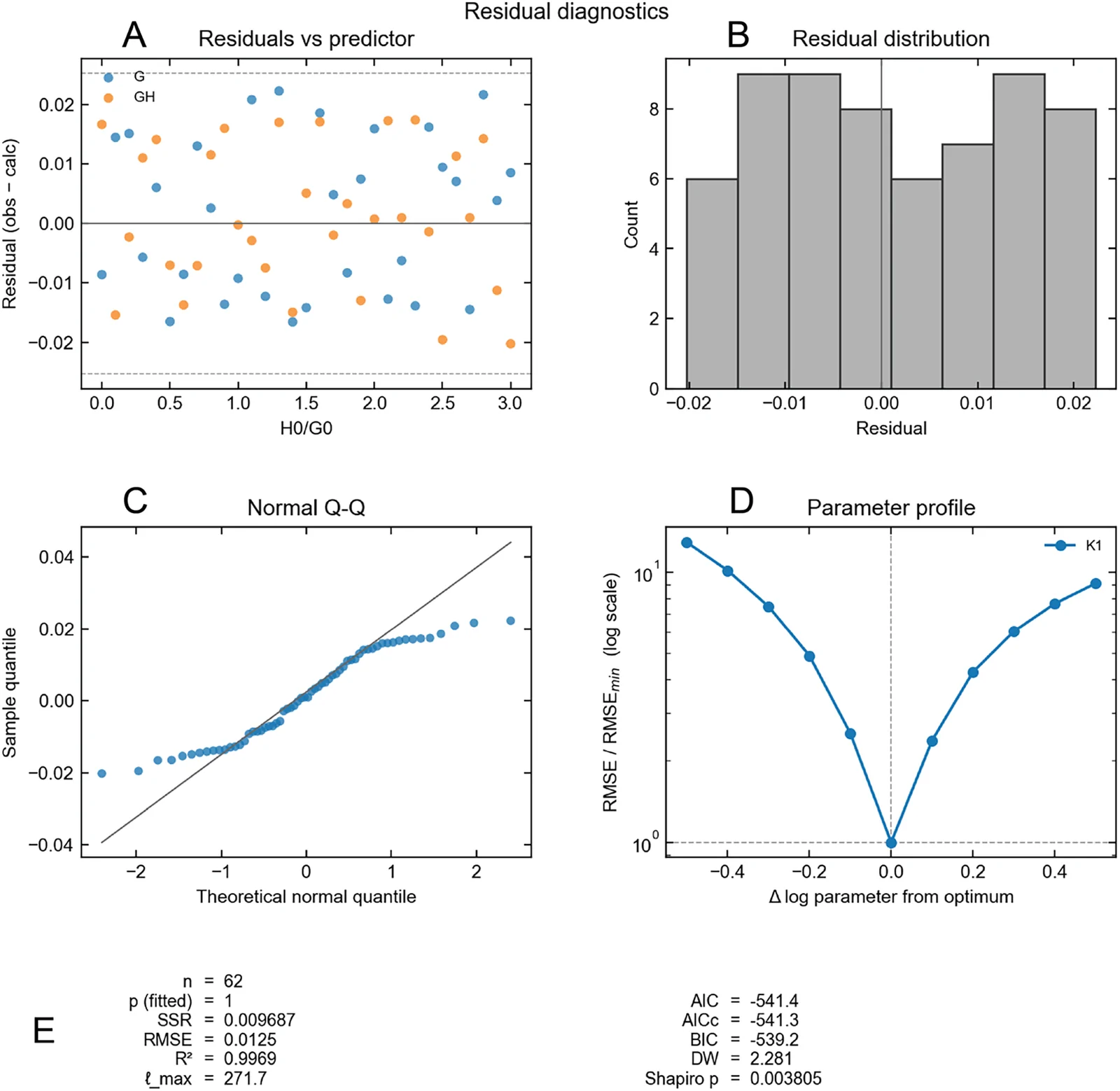
Four-panel residual diagnostic output for a 1:1 host–guest binding process with a binding constant *K*. (A) Residuals (in mM) plotted against *H*_0_/*G*_0_ for the two tracked species; dashed lines mark the ±2 × RMSE band. (B) Histogram of all 62 residuals. (C) Normal Q–Q plot of the residuals against standard-normal quantiles, with the diagonal reference line in gray. (D) 1D RMSE profile of log *K* over Δlog *K* ranging from −0.5 to +0.5. (E) Statistical descriptors.

A two-dimensional profile diagnostic (an extension of Musketeer’s 1D profile) diagnoses pairwise parameter correlation. Two parameters are pinned simultaneously over an *n* × *n* grid, with each axis spanning around the joint optimum; every other free parameter is refit at each grid point, and the resulting RMSE matrix is rendered as a colormap with iso-RMSE contours overlaid at 1.1, 1.5, 2, and 5 RMSE_opt_ values. Long diagonal valleys are the visual signature of strong pairwise correlation: each parameter can absorb the other’s perturbation, so the joint likelihood surface is an extended banana-shaped trough rather than a single well. Round wells indicate that the two parameters are independently identified.

In addition, a novel local sensitivity test is tentatively proposed here. For a user-selected subset of *N* variables (any combination of equilibrium or rate constants, concentrations and volumes), the RMSE is evaluated on a 3^*N*^ grid around the fit anchor (each variable at {−δ, 0, +δ} in log space, δ being defined by the user) and a paired-difference average parameter ξ_*X*_ is computed for every selected variable *X* over the 2·3^*N*–1^ pair-differences in which *X* is perturbed by ±δ while the other *N* – 1 variables run through all 3^*N*–1^ configurations of their levels (see eq 17). Because any combination of variables can be selected, the user can probe coupling between fitted parameters, between held-fixed quantities, and across the two (for example, asking how much of the apparent precision in a log *K* depends on the assumed accuracy of a stock concentration).

17 $$\xi_{X}=\frac{1}{2•3^{N-1}}\sum_{\delta X\in \left\{-\delta ,+\delta \right\}}\sum_{\mathrm{others}}\frac{\mathrm{RMSE}\left(X_{\mathrm{opt}}+\delta X,\mathrm{others}\right)-{\mathrm{RMSE}}_{\mathrm{opt}}}{{\mathrm{RMSE}}_{\mathrm{opt}}}$$

Equilibrist proposes three additional Hessian-derived diagnostics:

(a) A parameter correlation heatmap that displays the normalized, color-coded off-diagonal elements of the Hessian-derived covariance matrix **∑** (see eqs 6 and 18).
18 $$\rho_{\mathrm{ij}}=\frac{\sigma_{\mathrm{ij}}}{\sqrt{\sigma_{\mathrm{ii}}\sigma_{\mathrm{jj}}}}$$
  ρ_*ij*_ ∈ [−1, +1] thus reports the correlation between fitted parameters θ_*i*_ and θ_*j*_ at the best-fit optimum; ρ_*ij*_ = 0 indicates that the two parameters are independently determined. Strong off-diagonal correlations (|ρ_*ij*_| close to 1) are a warning that the Hessian standard errors of eq 7 might conceal a pairwise degeneracy. A classic example of such degeneracy in supramolecular chemistry is the *K*_1_ and *K*_2_ anticorrelation in a 1:2 binding model when the data do not sufficiently constrain the two stepwise binding events independently.^11^
(b) A sloppy-spectrum^26,27^ panel that performs an eigen decomposition of the covariance matrix **∑**. Each eigenvector **v**_*i*_ is a linear combination of the fitted parameters, and the associated standard deviation σ_*i*_ is the uncertainty along that direction. Sorting σ_*i*_ from largest to smallest yields the sloppy spectrum: the largest σ_*i*_ identify the sloppy directions (i.e., combinations of parameters along which the data are nearly insensitive, and the joint likelihood surface is flat), while the smallest σ_*i*_ identify the stiff directions, which the data constrain tightly. Equilibrist also reports a per-parameter impact ranking computed using the conditional standard error σ_alone_(*k*) (see eq 19) obtained when all other parameters are held fixed at their best-fit values. This allows the user to identify which of the fitted parameters dominate the sloppy combinations.
19 $$\sigma_{\mathrm{alone}}\left(k\right)=\sqrt{\frac{2\sigma^{2}}{H_{\mathrm{kk}}}}$$
(c) A parameter significance (*t* test) panel that reports, for each fitted parameter θ_*k*_, the t-statistic *t*_*k*_ (see eq 20), where σ­(θ_*k*_) is the marginal Hessian error of eq 7 and θ_*k*,0_ is a user-set reference (default 0). Under Gaussian residuals, *t*_*k*_ follows a Student t-distribution with (*n* – *p*) degrees of freedom: *p* < 0.05 means θ_*k*_ is statistically distinguishable from θ_*k*,0_, while a large *p*-value indicates a fitted estimate that the data do not actually require.
20 $$t_{k}=\frac{\theta_{k}-\theta_{k,0}}{\sigma \left(\theta_{k}\right)}$$

Equilibrist then proposes a bootstrap confidence interval (CI) generator^28,29^ that does not rely on the Hessian. At each bootstrap iteration, a synthetic data set y* is generated from the best-fit calculated values *y*_*i*_ by injecting residuals *r*_i_^*^ resampled (with replacement) from the original residual vector {*r*_1_, ..., *r*_n_}. The full fit is rerun on each bootstrap data set, yielding an empirical distribution for every fitted parameter from which percentile CIs are extracted. The bootstrap replicates are dispatched with a live progress bar (a 500-iteration bootstrap on a typical equilibrium fit completes in a few minutes or faster on a laptop).

Equilibrist offers also a jackknife (leave-one-out) cross-validation generator. Each experimental observation (single concentration, full NMR spectrum, or full UV–vis spectrum) is dropped in turn, the fit is repeated on the remaining points, and the standard deviation is reported.^30,31^

Finally, Equilibrist offers a Monte Carlo generator that propagates user-specified experimental input uncertainties (concentrations of stock solutions, titrant volumes, etc.) into the fitted parameters. This is conceptually distinct from the bootstrap, which holds the input quantities fixed and resamples the residuals: here, the user assigns a relative uncertainty to each input, those inputs are redrawn from their assigned distributions at every iteration, and the full fit is repeated on each draw. The empirical spread of the resulting parameter samples isolates the contribution of input uncertainty to parameter uncertainty and complements the standard errors in eq 7. In practice, input uncertainty can dominate errors for many supramolecular titrations, and reporting a Hessian standard error or a bootstrap CI in isolation can give a misleading impression of precision.^4,32^

## Implementation

### Architecture

Equilibrist is implemented in Python 3.9+ and is organized into a set of independent modules. The user interface is provided by Streamlit, which compiles the Python source code into a reactive web application that runs locally and is accessed through a standard web browser. The module structure separates tasks as follows: the parser (Equilibrist_parser.py) is responsible for interpreting the script and producing a structured representation; the network builder (Equilibrist_network.py) constructs the stoichiometric data structures required by the solvers; individual fit modules (Equilibrist_fit_conc.py, Equilibrist_fit_nmr.py, Equilibrist_fit_spectra.py, Equilibrist_kinetics.py, Equilibrist_kinetics_nmr.py, Equilibrist_kinetics_spectra.py) implement the fitting logic for each combination of experiment type and data type; and the main application file (app.py) assembles these into the interactive interface. Two additional modules implement the statistical validation layer: Equilibrist_diagnostics.py computes the information criteria, residual statistics, the four-panel diagnostic figure, two nonlinear identifiability tools (1D and 2D RMSE profiles), three Hessian-derived diagnostics (parameter-correlation heatmap, sloppy spectrum with per-parameter impact ranking, per-parameter *t* tests), and our local sensitivity test. Equilibrist_bootstrap.py provides mode-specific routines (bootstrap, jackknife (leave-one-out), and Monte Carlo input uncertainty propagators).

### Choice of Framework

Equilibrist’s interface is built on Streamlit rather than a native-desktop toolkit such as Qt (used by SupraFit),^9^ and this choice involves real trade-offs. A Streamlit application reexecutes its Python script in full on each user interaction, recomputing every cached intermediate that depends on a changed widget; for problems solved by Equilibrist, this time limitation is not significant. Streamlit was favored as the application can be deployed to any modern browser without compilation or operating-system-specific installers, which lowers the barrier for the principal target audience (chemistry graduate students and senior undergraduates).

### Script Language

The Equilibrist scripting language is a plain-text language organized into named sections introduced by a $ keyword. Table 1 summarizes the available sections.

**Table 1:** Summary of Equilibrist Script Sections

section	purpose	availability
$concentrations	initial species concentrations	required
$volumes	cell volume *V*_0_	required
$titrant	titrant identity/ies and concentration(s); solid mode supported	required for titrations
$reactions	chemical reactions with initial equilibrium and/or rate constant values, and optional soft bounds	required
$variables	algebraic combinations of species for plotting and/or NMR signal assignments	optional
$plot	*x*- and *y*-axis legends and ranges	optional
$NMR	NMR mode declaration (with specific instructions for fast, slow, and hybrid exchange regimes)	optional
$spectra	UV–vis/fluorescence mode declaration; optionally declares transparent species, the path length, and spectra to read from the input file	optional
$constraints	absolute and/or interparameter constraints	optional
$temperature	temperature used to link free Gibbs energies and free energies of activation to equilibrium and rate constants	optional

### Export Functions

All numerical results are exportable as a Microsoft Excel workbook containing at least three tabs: (1) the fitted data overlaid with the theoretical curves, (2) a copy of the script, and (3) a script with parameter values updated to the fitted results that can be copied back into the script editor as a starting point for a refined analysis. A fourth tab in the $spectra mode lists the extrapolated extinction coefficients of pure species listed in $reactions. Snapshots of concentration plots can be taken at any time, and high-quality figures can be downloaded as PDF vector graphics directly from the interface. Figure filenames are automatically generated from the script filename with a timestamp. To support FAIR^33,34^ (findable, accessible, interoperable, reusable) data-sharing practice and full reproducibility, Equilibrist exports a self-contained JSON session file that captures the complete state of an analysis: the script, the original experimental input, every fitted parameter with its Hessian standard error, the uncertainty estimates from any of the bootstrap, jackknife, and Monte Carlo layers that were run, the residual diagnostics, the seed used by the random-number generator, and the version strings of Python, NumPy, SciPy, and Equilibrist itself. The JSON file can be reimported to reconstruct the data and the analysis state. Together these features mean that an Equilibrist analysis can be archived as a single file deposited alongside the manuscript and can be reexecuted by an independent reader on a different machine.

## Results and Case Studies

The simplest test case (see Figure 1) is the titration of a guest **G** (initial concentration *G*_0_ 1.0 mM in a 0.50 mL sample) with a stock solution of a host **H** (10 mM) to form host–guest complex **GH** in a 1:1 stoichiometry. The binding affinity *K* and concentrations can be modified at will; the impact of the changes on the concentrations of each species can be observed in real time in the main plot area. The user may also upload experimental data (a Microsoft Excel sheet with volumes of added titrant, and concentrations of a selection of species throughout the titration, see data points in Figure 2A,B before and after the fit, respectively). Users should also note features (a)–(e) in Figure 1: (a) the initial concentration of guest in the analyte has a required “0” suffix that differentiates the total concentration of guest *G*_0_ from the free guest concentration *G* during the titration; (b) the volume of the analyte solution can be provided in L, mL or μL (written “uL”); (c) the concentration of titrant solution has a required “t” suffix to differentiate it from the total concentration of host (free and bound) *H*_0_ added to the analyte, and the concentration of free host *H*; (d) in the equilibrium between free guest **G** and host **H** and their complex **GH**, the “=” sign indicates an equilibrium; (e) the main plot covers the range of 0–3 equiv of host relative to the total guest concentration *H*_0_/*G*_0_.

The four-panel residual diagnostic figure produced by Equilibrist (62 concentrations across 31 *H*_0_/*G*_0_ ratios, *p* = 1 fitted parameter log *K*) is shown in Figure 2. Figure 2A plots the residuals as a function of the *H*_0_/*G*_0_ ratio for the two tracked species (**G** in blue, **GH** in orange): the points scatter randomly, with no systematic trend across the titration profile, between the two species, or as a function of titrant addition, consistent with a correctly specified 1:1 binding model. The histogram of all 62 residuals (Figure 2B) is roughly symmetric and centered on zero but flat-topped rather than peaked; the feature also shows in the normal Q–Q plot (Figure 2C), where the residuals follow the reference line in the center and then flatten in both tails. The Shapiro–Wilk test (*p* = 0.0038) confirms this departure from normality. Figure 2D shows the one-dimensional RMSE profile of the single fitted parameter log *K*: a steep V-shaped valley in which RMSE rises by 13-fold within ±0.5 log unit of the optimum, indicating that log *K* is tightly identified by the concentration data.

The cell may contain multiple species (see concentrations *A*_0_, *B*_0_ and *C*_0_ in Figure 3), and titrations can also be carried out upon addition of aliquots of a solid (see feature *a* in Figure 3). That solid can be a mixture of species, entered as molar ratios (see feature *b*; *C*/*A* ratio 1:0.5, or 67:33). Instead of entering equilibrium or rate constants, the user may also enter free Gibbs energies Δ*G* or free energies of activation Δ*G* ^‡^ for a given reaction, in kcal/mol (see feature *c*). Those are then converted to equilibrium constants *K* and rate constants *k* using eqs 21 and 22 (Van’t Hoff and Eyring relations, assuming elementary steps, respectively; *k*_B_ is the Boltzmann constant and *h* the Planck constant). The default temperature is 25 °C, but it can be changed with the section heading $temperature.

21 $$K=e^{-\Delta G/\mathrm{RT}}$$

22 $$k=\frac{k_{B}T}{h}e^{-\Delta G^{‡}/\mathrm{RT}}$$

A key feature of Equilibrist allows the user to define any variable as functions of concentrations, or as functions of other variables (see section $variables in Figure 3). In feature *d*, variable S is defined as the sum of the concentrations of species **A**, **B**, **C**, and **D**. Ratios (%A, for example) can then be defined as shown in feature *e*. Any concentration(s) or variable(s) can be plotted on the *y*-axis (see $plot section and Figure 3A). Statistics of the fit are provided, with objective evaluations being the number of iterations required to reach the tolerance threshold (adjustable in the software); optimized constants and their corresponding free energy terms are listed as well, with their associated errors (see Figure 3B).

**Figure 3:**
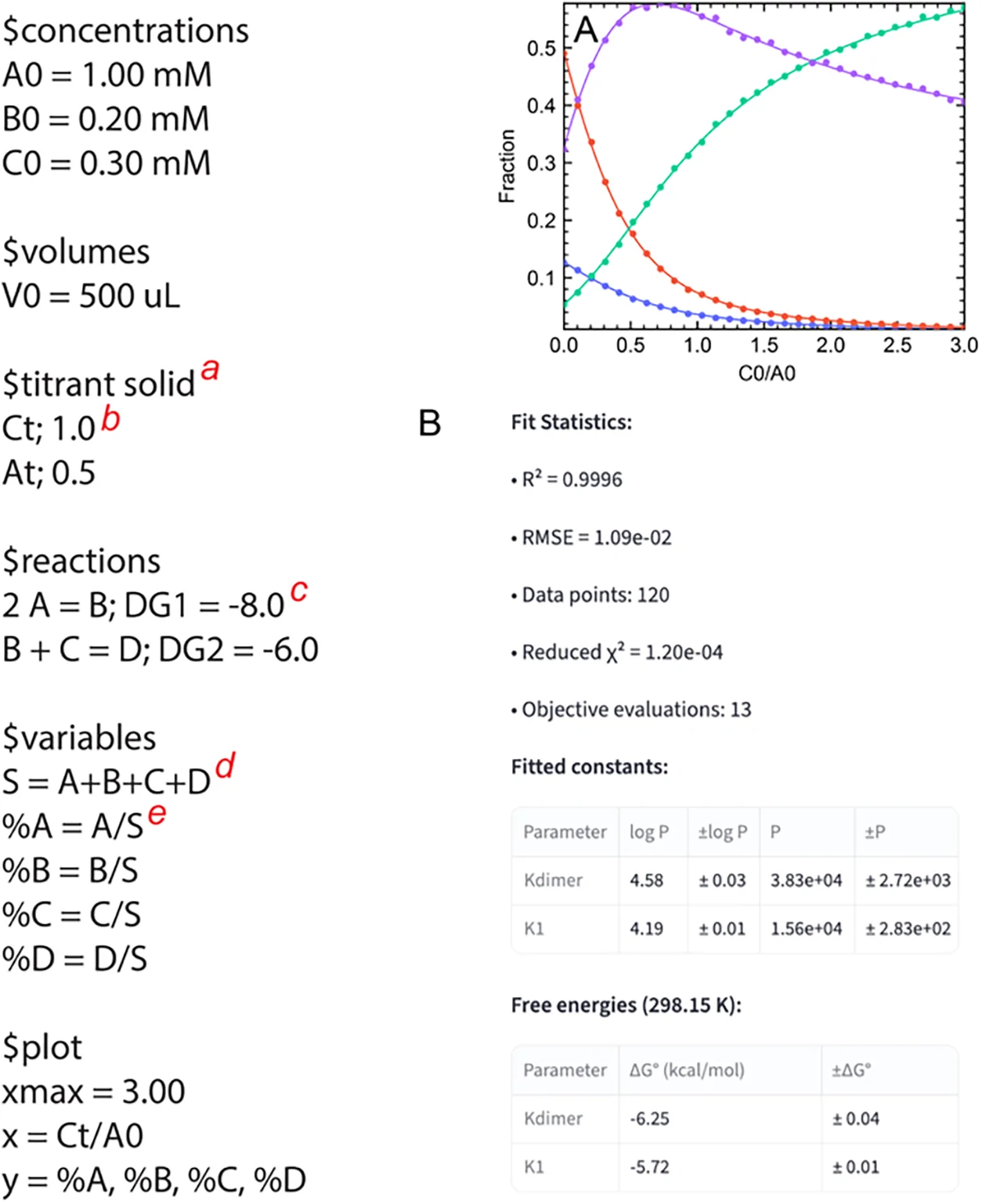
Equilibrist script for a titration with a mixture of solid species that highlights the $variables section. (A) Plot generated by Equilibrist after fitting experimental data: fraction of species **A** (in blue), **B** (in red), **C** (in green), and **D** (in violet). (B) Statistical parameters associated with the fitting process; fitted constants with associated errors, and their conversion into free energy terms.

Equilibrist can import NMR data (chemical shifts and/or signal integrations) when the $NMR heading is present in the script. Figure 4 shows a 2:1 host–guest binding process, with binding constants *K*_1_ and *K*_2_, that is fast on the NMR time scale. Chemical shifts of the guest signals are thus a population-weighted average of the chemical shifts of the free species **G**, the 1:1 complex **GH** and the 2:1 complex **GH2** (see eq 8); the sum of the concentration of these species is defined as *G*_tot_ in $variables (see feature *a* in Figure 4), and invoked in the $NMR section after the keyword “shift” that specifies a fast exchange regime; similarly, *H*_tot_ is defined in $variables, and called in the $NMR section. The input file (Figure 4C) contains the volume of added titrant solution and the chemical shifts, in ppm, corresponding to the guest signals in species **G**, **GH**, and **GH2** (*G*_tot_), and to the host signals in species **H**, **GH**, and **GH2** (*H*_tot_). Concentrations of all species are plotted in Figure 4A after fitting (free guest **G** in blue, free host **H** in red, 1:1 complex **GH** in green, and 2:1 complex **GH2** in violet); Figure 4B shows the fitting of the chemical shifts (*G*_tot_ in blue, *H*_tot_ in red).

**Figure 4:**
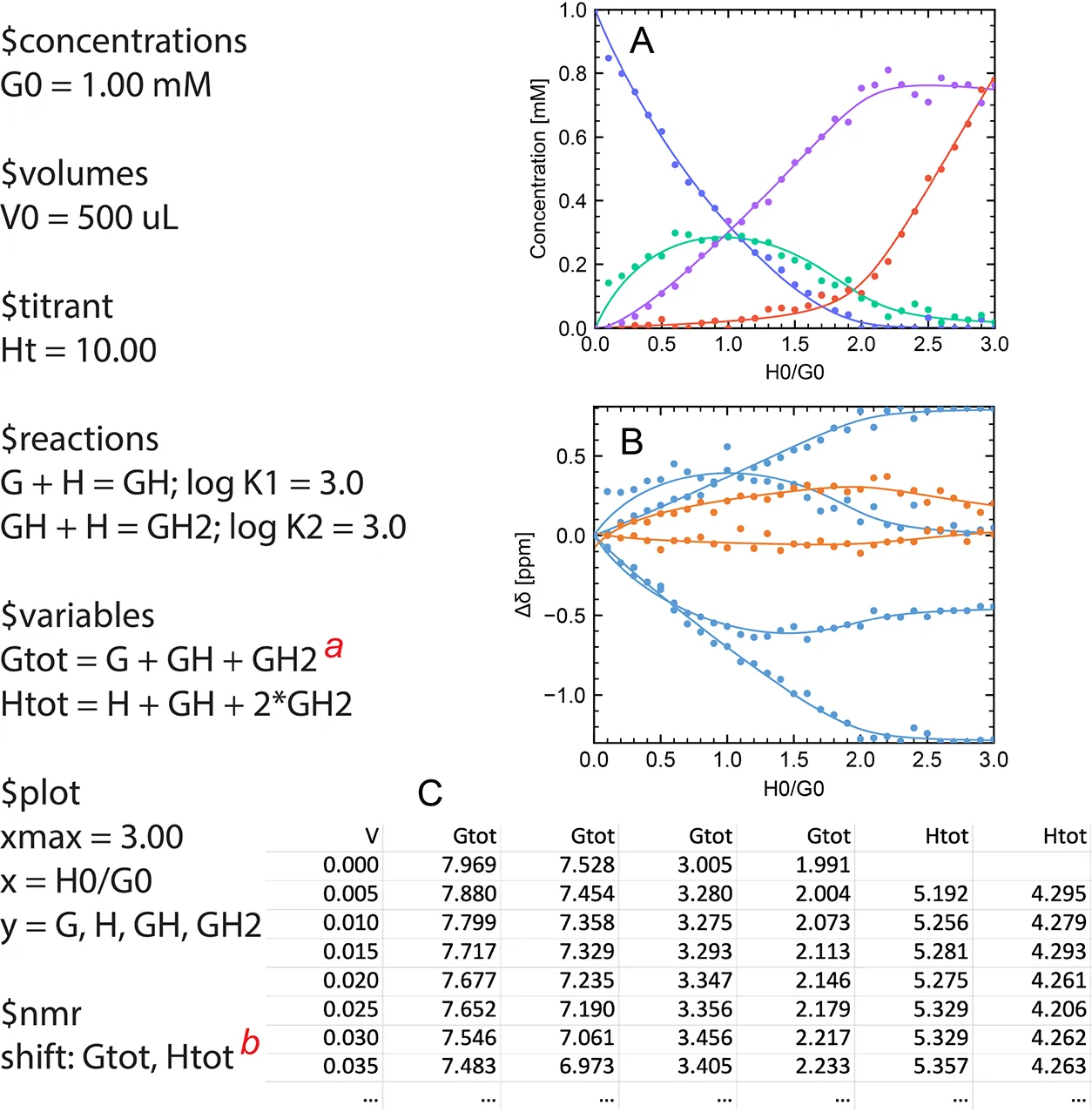
Equilibrist script for a 2:1 host–guest binding process that is fast on the NMR time scale, with binding constants *K*_1_ and *K*_2_, using NMR chemical shifts as input. (A) Plot generated by Equilibrist, with concentrations calculated from chemical shifts (species **G**, **H**, **GH**, and **GH2** in blue, red, green, and violet, respectively) as a function of *H*_0_/*G*_0_. (B) Changes in chemical shifts for the 6 signals tracked in (C) (guest signals in blue, host signals in red); (C) excerpt of the input file; volume of added titrant solution in the first column [mL], chemical shifts observed for guest signals (next 4 columns) and host signals (next 2 columns).

The fit converged in 51 iterations to log *K*_1_ = 4.60 and log *K*_2_ = 4.68. Durbin–Watson *d* = 1.79 confirms uncorrelated residuals, but Shapiro–Wilk *p* = 2.5 × 10^–8^ clearly rejects normality. The 2D RMSE profile (see Figure 5) shows that log *K*_1_ and log *K*_2_ remain independently identified (they round well) despite broad confidence ellipses. The three uncertainty methods diverge as a result. The Hessian standard errors and the jackknife agree closely (the standard error from jackknife is 0.42 and 0.40 for log *K*_1_ and log *K*_2_, respectively; the Hessian 2D ellipse covers the same range, see Figure 5). The bootstrap percentile CI (*B* = 500) is noticeably tighter and slightly asymmetric: log *K*_1_ ∈ [+4.04, + 5.09], log *K*_2_ ∈ [+4.25, + 5.34] at 95% confidence, corresponding to bootstrap σ = 0.27 and 0.28, respectively, approximately 65% of the Hessian/jackknife width. Our local sensitivity test on log *K*_1_ and log *K*_2_ (δ = 0.2 log unit) returns ξ = 0.33% and 0.28%, respectively, illustrating the rather flat RMSE surface around the optimum; conditional standard errors σ_cond_ = 0.18 and 0.20 are comparable to the bootstrap σ. With Shapiro–Wilk rejecting normality, the bootstrap percentile CI should be the uncertainty to report: it directly captures the empirical sampling distribution of the fitted parameters. Even at the tighter bootstrap intervals, the two binding constants overlap substantially: the small difference (Δlog *K* = 0.08) is within sampling noise and should not be overinterpreted as a chemically meaningful difference between the two binding events.

**Figure 5:**
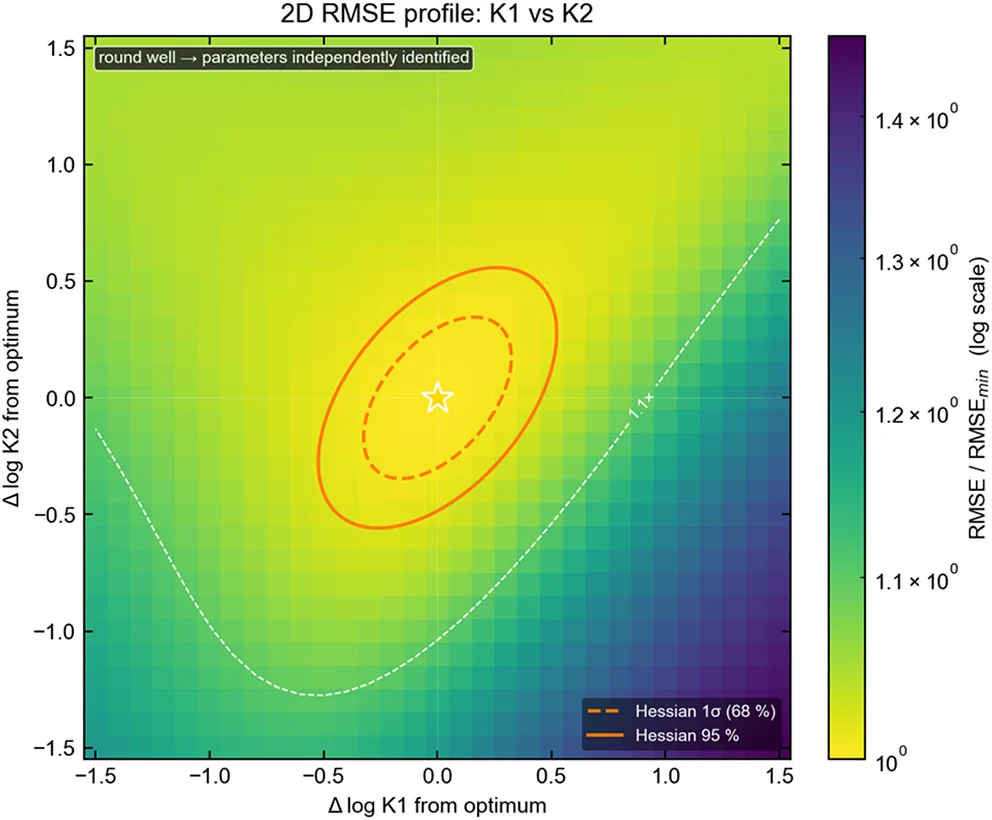
2D RMSE profile of log *K*_1_ and log *K*_2_ for the 2:1 host–guest binding process (see Figure 4 for details). Each pixel is the model’s RMSE at the indicated (Δlog *K*_1_, Δlog *K*_2_) perturbation from the best-fit optimum (31 × 31 grid); the color encodes RMSE/RMSE_opt_ on a logarithmic scale. The orange contours mark the Hessian-derived confidence regions at 1σ (68%, dashed) and 95% (solid). The white dashed curve traces the 1.1 × RMSE_opt_ iso-contour, demarcating the region in which the fit quality remains within 10% of its best value.

Figure 6 shows the formation of a 3:1 host–guest complex, in which the formation of the 1:1 and 2:1 complexes is fast on the NMR time scale, but the last complexation with the third host is slow. The user monitors the growth of two of the 3:1 complex signals, that are assigned to 1 and 3 NMR-active nuclei of the guest (^1^H, ^19^F, etc.); they also monitor the diminishing integration of only one of the population-averaged signals of the free guest, its 1:1 complex and its 2:1 complex that exchange rapidly (assigned to 1 NMR-active nucleus in the guest structure; see Figure 6C for a summary); variable G012 is defined as the sum of the concentrations of species **G**, **GH**, and **GH2** (see feature *a* in Figure 6). Finally, the user monitors changes in chemical shifts of three other population-averaged **G** + **GH** +** GH2** signals (they could have integrated all 4 signals corresponding to **G** + **GH** + **GH2**, but the example shows this is not necessary for Equilibrist to extract equilibrium constants). In the $NMR section, “integration: 1, 3, 1” (see feature *b*) indicates that signal integrations correspond to 1×, 3×, and 1× NMR-active nuclei, respectively, in species **GH3**, **GH3**, and fast-exchanging G012 (see excerpt of input file in Figure 6E; note that the volume of added titrant is always in the first column, followed by integrations if available, and finally by the chemical shifts; the order must be respected). Concentrations of species **G**, **H**, **GH**, **GH2**, and **GH3** are shown in blue, red, green, violet, and cyan, respectively, as a function of *H*_0_/*G*_0_ (see Figure 6A). Variable G012 has two closely overlapping orange traces, one calculated from signal integration and one from chemical shifts.

**Figure 6:**
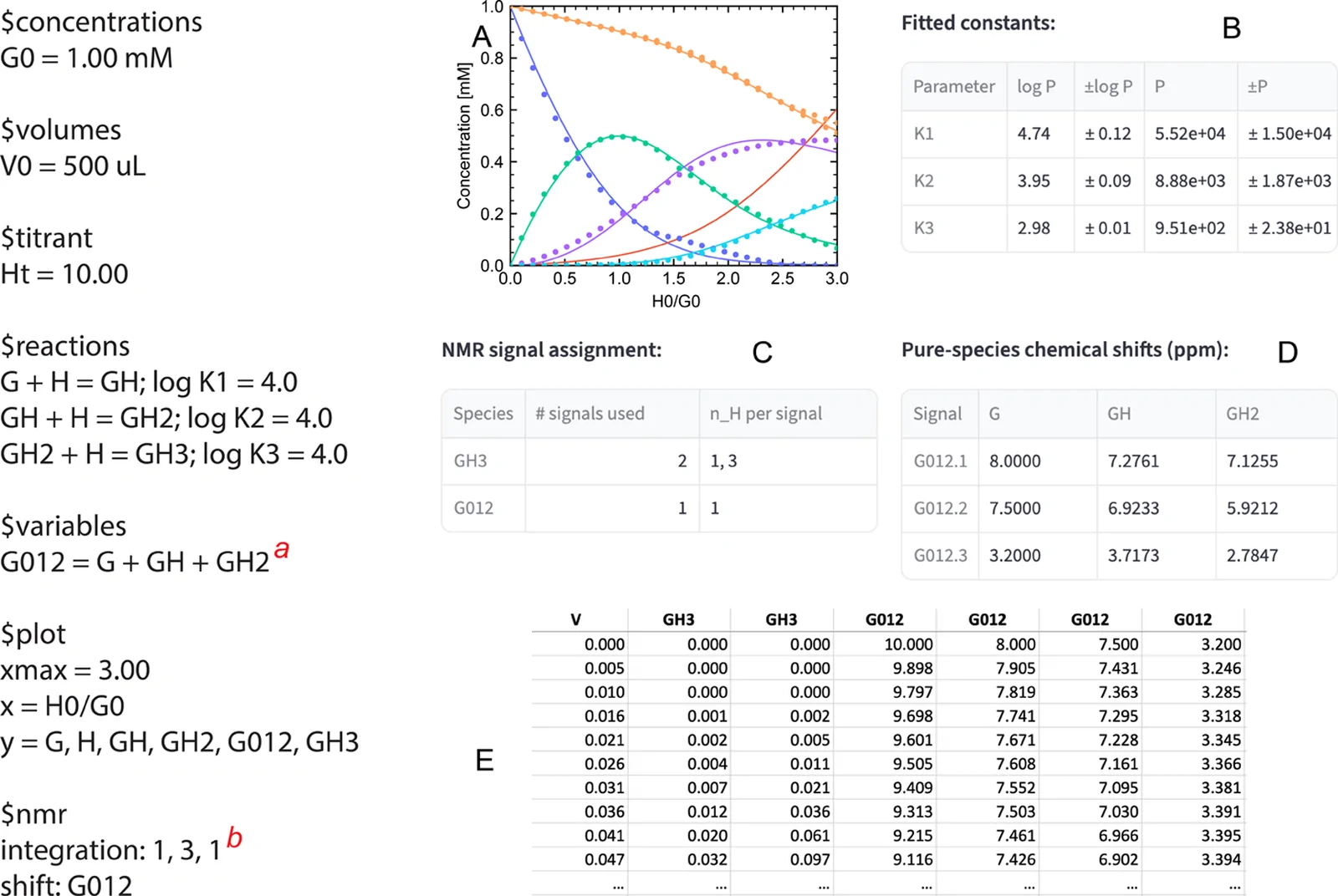
Equilibrist script for a 3:1 host–guest binding process, with binding constants *K*_1_–*K*_3_; free guest **G**, its 1:1 complex **GH**, and its 2:1 complex **GH2** exchange fast on the NMR time scale; formation of the 3:1 complex **GH3** is slow on the NMR time scale. (A) Plot generated by Equilibrist, with concentrations calculated from chemical shifts and integrations (species **G**, **H**, **GH**, **GH2**, and **GH3** in blue, red, green, violet, and cyan; sum of concentrations G012 in orange). (B) Fitted constants with errors. (C) Summary showing that species **GH3** is quantified with 2 signals corresponding to 1 and 3 NMR-active nuclei, and **G** + **GH** + **GH2** with 1 signal corresponding to 1 nucleus; (D) extrapolated chemical shifts of the pure species nuclei; (E) excerpt of input file; volume of added titrant solution in the first column [mL], signal integrations (next three columns) and chemical shifts (next 3 columns).

Equilibrist has all necessary features needed for acid–base titrations, including the fitting of p*K*_a_’s. Let us consider, for example, the titration of phosphoric acid (1.0 mM) with a mixture of sodium hydroxide and an organic amine (10 mM and 5.0 mM in the titrant stock solution, respectively, see Figure 7, script A). The unitary activity of water is entered as [H_2_O] = 1 “M” (see feature a), and *K*_w_ is the ionic product of water (feature b). The pH must be defined as a variable (note the 1000-fold correction in feature c, as default concentration units are mM). While script A is fully functional, Equilibrist accepts a more efficient version if the keyword “acid–base” is called after $reactions (see feature d, Figure 7, script B). The activity of water and its ionic product are no longer needed, and acid–base reactions are replaced with a list of acids and their conjugate bases, followed by their p*K*_a_ (see feature e). Under $plot, “ylabel” can be used to customize the y-label legend (feature *f*). In this example, the p*K*_a_ of the tertiary ammonium is allowed to fit (see Figure 7C,D).

**Figure 7:**
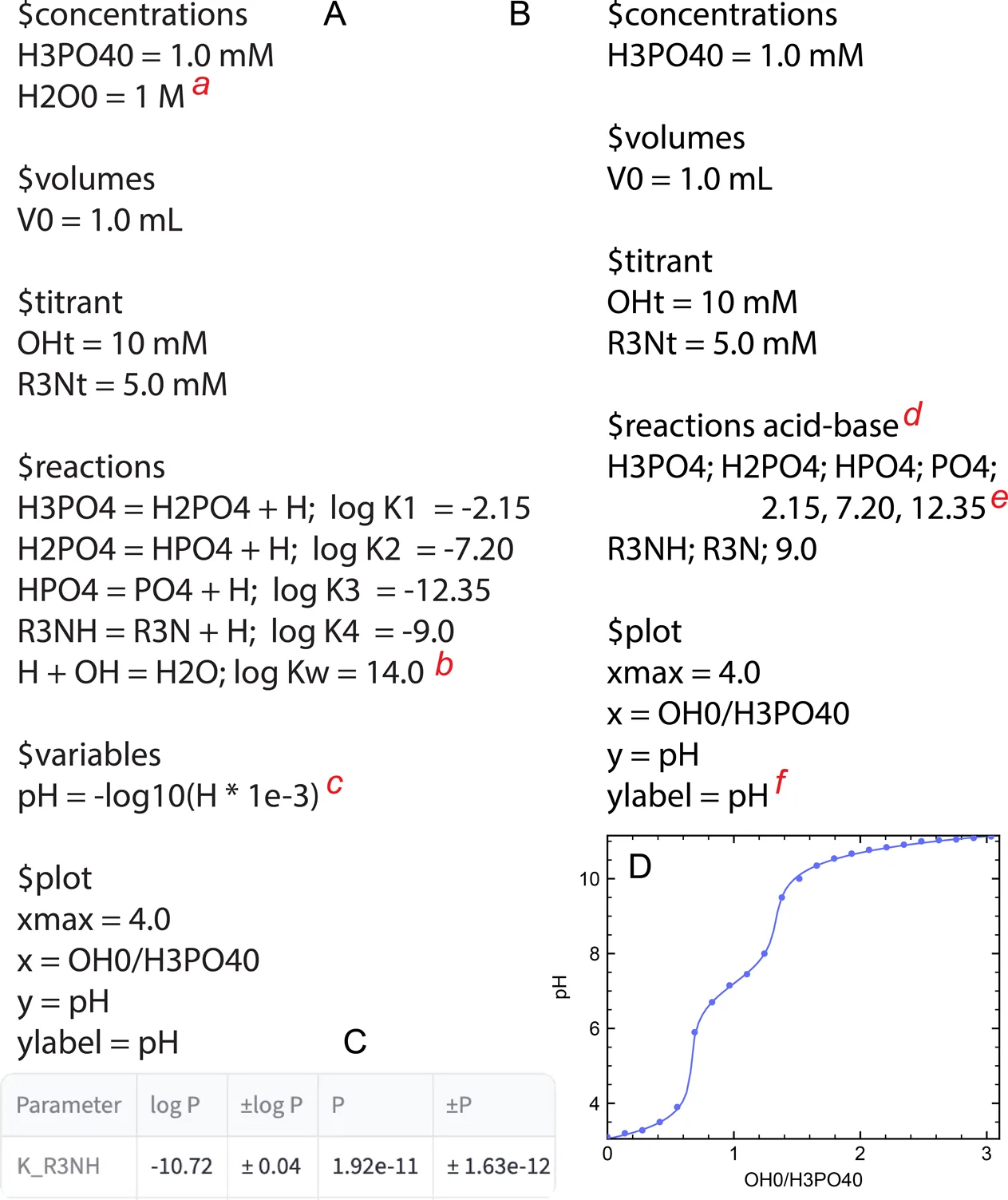
Equilibrist scripts for the acid–base titration of phosphoric acid with a 1:2 mixture of hydroxide and a tertiary amine (R_3_N). (A) Original syntax. (B) Simplified version when the keyword “acid–base” is added to $reactions. (C) Optimized p*K*_a_ of the tertiary ammonium cation is 10.72 (±0.04 – Hessian standard error). (D) pH as a function of the number of equivalents of hydroxide relative to phosphoric acid.

Equilibrist allows the determination of both equilibrium and rate constants. To trigger the kinetic mode (see Figure 8), the user defines at least one irreversible reaction using the “>” sign instead of the equilibrium “=” sign in the $reactions section, or at least one equilibrium defined with the symbols “<>”, with forward and reverse reaction rate constants (see feature *a* in Figure 8). Instantaneous preequilibria are still defined with the “=” symbol and equilibrium constants. In kinetic mode, the $concentrations section lists the concentrations of species at *t* = 0 (in mM unless specified otherwise). The units of rate constants include molar and seconds (for example, *k*_1_ in M^–2^ s^–1^ and *k*_–1_ in M^–1^ s^–1^ in the Figure 8 reaction); the *x*-axis of the main plot is the time in seconds (see Figure 8A). Free energy terms (Figure 8C) are obtained from the fitted constants (Figure 8B); the user should interpret the free energies of activation cautiously, as they are strictly meaningful only if the fitted rate constants correspond to single elementary reaction steps (see feature *b*).

**Figure 8:**
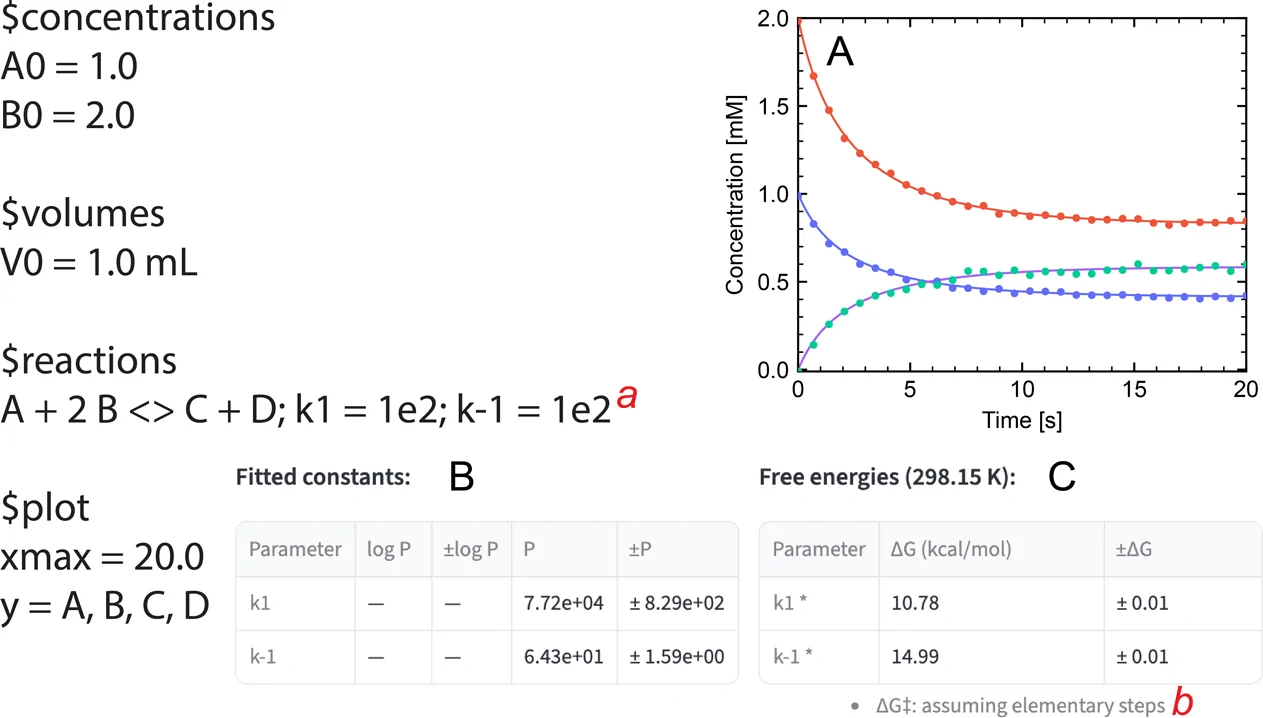
Equilibrist script that triggers the kinetic mode. (A) Concentrations of species **A** (blue trace), **B** (red trace), **C** (green data points), and **D** (violet trace) as a function of time [s]; concentrations of species **C** and **D** are equal and traces overlap. (B) Fitted rate constants and their errors. (C) Free energies of activation calculated with the Eyring equation, eq 22, assuming elementary steps.

In both titration and kinetic modes, when the $spectra heading is invoked (see Figure 9), Equilibrist can import series of UV–vis/circular dichroism/fluorescence spectral data used to monitor the reactions and grouped in a Microsoft Excel sheet (with the first column being the added volume of titrant or time, and the next columns showing absorbances/ellipticities/emission intensities at different wavelengths). The software offers several features, such as auto-optimizing the range of wavelengths to minimize the error on the constants, allowing or not negative absorbances (for circular dichroism monitoring, for example), reading spectra of pure species from the input file, and allowing the specification of transparent species that force extinction coefficients to 0 at all wavelengths (see feature *a* in Figure 9; or by extension, nonemissive species; both keywords “transparent”: or “non-emissive”: can be used interchangeably); the path length is 1 cm by default, but can be changed with the variable “path” in the $spectra section. Spectra of pure species can also be provided in a second tab of the input file instead of being reconstructed upon fitting (see the keyword “read”, feature *b* in Figure 9). This example is also used to showcase the $constraints section (see feature *c*), in which any combination of constraints can be applied. Figure 9A features the extracted concentrations of all listed species in the $plot section at time *t* ranging from 0 to *x*_max_, Figure 9B shows the spectral data (from blue to red as titration or time progresses), and Figure 9C shows the calculated spectra of the pure species.

**Figure 9:**
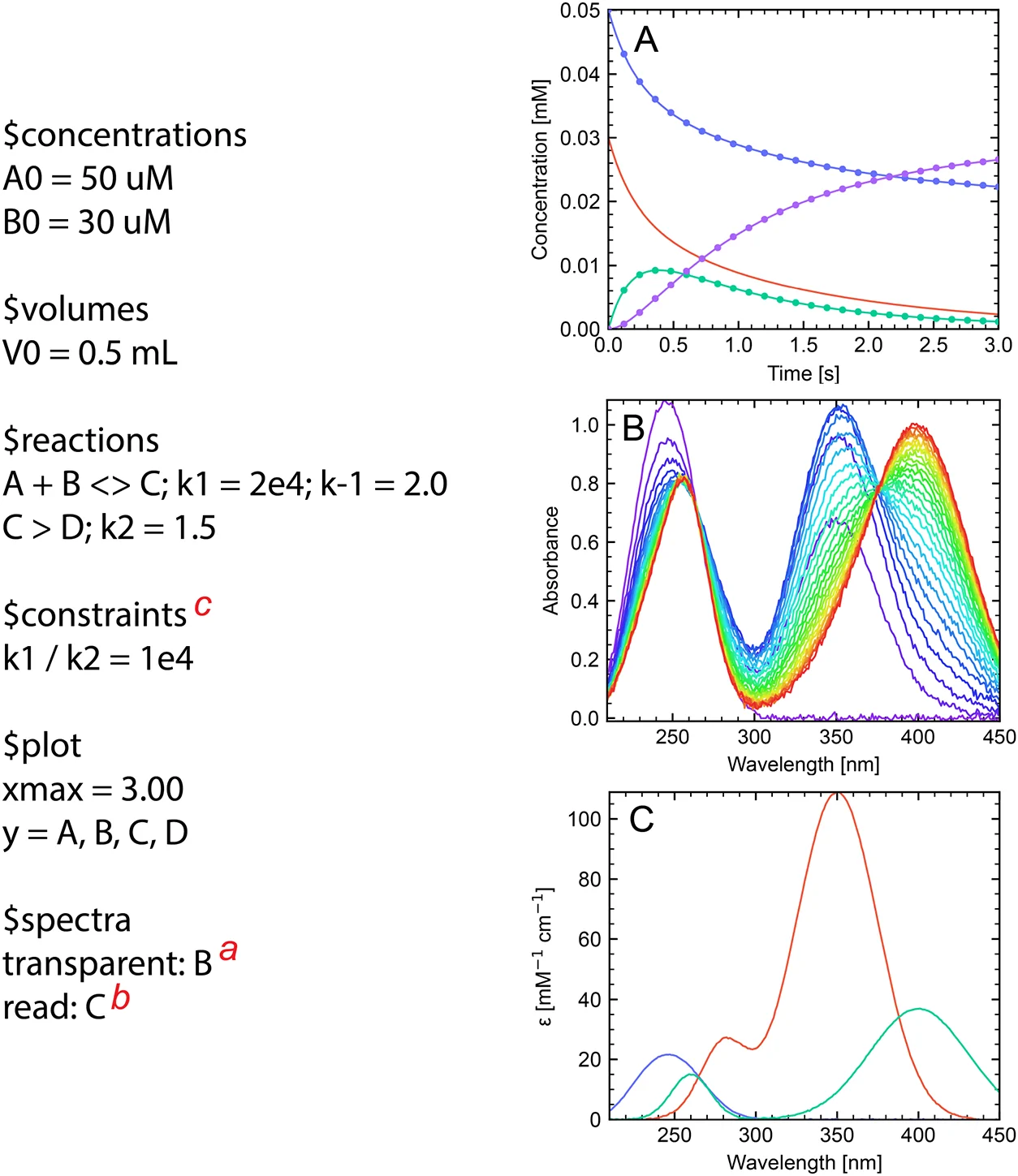
Equilibrist script that triggers the kinetic mode and reads UV–vis absorption spectra as input. (A) Concentrations of species **A** (in blue), **B** (in red), **C** (in green), and **D** (in violet) as a function of time [s]. (B) UV–vis spectra at various times (blue trace at *t* = 0, changing to red as the reaction progresses). (C) Computed spectra of pure species **A** (blue trace), **C** (red trace), and **D** (green trace).

The fit converged to *k*_1_ = 5.0 × 10^4^ M^–1^ s^–1^, *k*_–1_ = 0.98 s^–1^ and *k*_2_ = 2.0 s^–1^. We use this example to feature two Hessian-derived diagnostics: (1) the correlation heatmap (Pearson ρ from the Hessian-derived covariance, see Figure 10A) returns ρ­(*k*_1_, *k*_–1_) = +0.80, ρ­(*k*_–1_, *k*_2_) = −0.80, ρ­(*k*_1_, *k*_2_) = −0.48; two of three off-diagonals exceed the |ρ| > 0.5 threshold at which Equilibrist flags a pair as worth visualizing in the 2D RMSE profile. The *k*_1_-vs-*k*_–1_ 2D profile (Figure 10E) shows a long valley extending predominantly along the *k*_–1_ axis with a modest diagonal tilt (an equal-and-opposite *k*_1_/*k*_–1_ pairing pointing to a *K* = *k*_1_/*k*_–1_ degeneracy would return a 45-degree diagonal). (2) The sloppy-spectrum panel (Figure 10B–D) shows the Hessian three eigendirections with σ_i_ ranging from 3 × 10^–4^ for v_1_ to 2 × 10^–3^ for v_3_, v_1_ being the stiffest, i.e., the best-determined linear combination (coefficients −0.77, + 0.23, + 0.59 for *k*_1_, *k*_–1_ and *k*_2_, respectively); v_3_ is the loosest, mostly aligned with *k*_–1_ alone (weight −0.97), and identifies *k*_–1_ as the parameter whose value is most weakly constrained (see Figure 10C).

**Figure 10:**
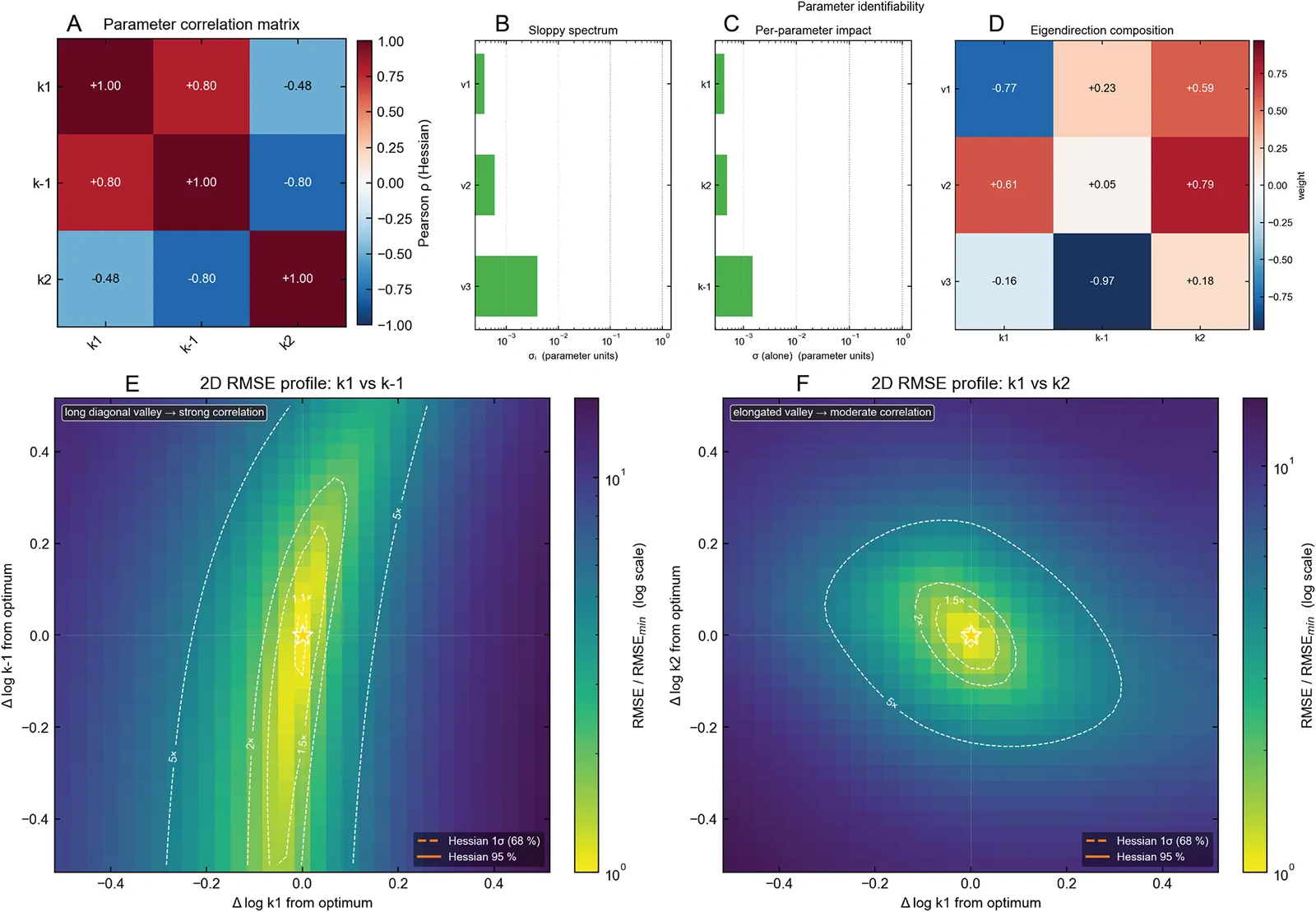
Diagnostics for the process A + B ⇌ C → D monitored by UV–vis spectroscopy; *k*_1_ = 5.03 × 10^4^ M^–1^ s^–1^, *k*_–1_ = 0.980 s^–1^, *k*_2_ = 1.99 s^–1^; *n* = 6266, *p* = 3. (A) Hessian-derived Pearson correlation matrix. (B) Sloppy spectrum. (C) Per-parameter ″alone″ σ (single-parameter Hessian standard error if the other two were known exactly), ranked by magnitude. (D) Eigendirection composition heatmap (rows = eigendirections, columns = parameters). (E) 2D RMSE profile of *k*_1_ vs *k*_–1_ in Δlog space. (F) 2D RMSE profile of *k*_1_ vs *k*_2_. The Hessian 1σ (68%) and 95% confidence ellipses (legend, bottom-right of panels (E) and (F)) are not visible because of the large *n*/*p* (2090) that shrinks the ellipses to <1% of the scan range.

The Monte Carlo propagator was set up with realistic experimental input uncertainties on the concentrations of species **A** and **B** (3%) and on the reference molar absorptivities (1%), and returned 95% CIs of [4.61 × 10^4^, 5.51 × 10^4^] M^–1^ s^–1^ for *k*_1_, [0.93, 1.01] s^–1^ for *k*_–1_, and [1.90, 2.08] s^–1^ for *k*_2_ i.e., relative uncertainties of 4.6%, 2.3%, and 2.4% respectively (Monte Carlo σ = 2.3 × 10^3^, 2.3 × 10^–2^ and 4.8 × 10^–2^ in their respective units). These CIs are dominated by input uncertainty, not residual noise: at *n*/*p* = 2090, the Hessian-derived σ on each rate constant is well below the Monte Carlo σ; reporting only the Hessian standard error would understate the experimentally limiting uncertainty by roughly an order of magnitude!

## Conclusions

Equilibrist allows users to assess the thermodynamics and kinetics of multicomponent systems. It provides a unified scripting language, a dual-method nonlinear optimizer, a flexible interparameter constraint system, and multiple fitting modalities covering the most common spectroscopic techniques employed in organic, inorganic, supramolecular, and biochemistry laboratories. Equilibrist features a statistical validation and identifiability layer. Information criteria (AIC, AIC_c_, BIC) with Akaike weights and a Fisher *F*-test handle model selection; four-panel residual plots with Durbin–Watson and Shapiro–Wilk tests flag least-squares violations. Three uncertainty layers (bootstrap, jackknife, and Monte Carlo input uncertainty propagation) return per-parameter σ and percentile CIs alongside the Hessian standard errors. Identifiability is mapped by a Pearson correlation heatmap, a per-parameter *t*-test, a newly introduced local sensitivity coefficient ξ, 1D and 2D RMSE profiles, and a sloppy-spectrum eigen decomposition with composition heatmap and impact ranking. Fits export to self-contained JSON sessions (script, SHA-256-hashed input, all outputs, diagnostics, seed, and environment versions), aligning Equilibrist with FAIR data-sharing recommendations expected for software-driven analyses in physical and analytical chemistry. The software is freely available and is designed to lower the barrier to rigorous quantitative analysis for a broad community of chemists and students at all academic levels.

## Data Availability

The code, manual, and tutorials are freely available at https://github.com/masson-group-ohiouniversity/Equilibrist (DOI: 10.5281/zenodo.18906724).
